# Breaking the Axis‐Symmetry of a Single‐Wall Carbon Nanotube During Its Growth

**DOI:** 10.1002/advs.202304905

**Published:** 2023-10-28

**Authors:** Lili Zhang, Ziwei Xu, Tian‐liang Feng, Maoshuai He, Thomas Willum Hansen, Jakob Birkedal Wagner, Chang Liu, Hui‐Ming Cheng

**Affiliations:** ^1^ Shenyang National Laboratory for Materials Science Institute of Metal Research Chinese Academy of Sciences 72 Wenhua Road Shenyang 110016 China; ^2^ School of Materials Science and Engineering Jiangsu University Zhenjiang 212013 China; ^3^ College of Chemistry and Molecular Engineering Qingdao University of Science and Technology Qingdao 266042 China; ^4^ DTU Nanolab Technical University of Denmark Fysikvej Kongens Lyngby 2800 Denmark; ^5^ Institute of Technology for Carbon Neutrality Shenzhen Institute of Advanced Technology Chinese Academy of Sciences 1068 Xueyuan Road Shenzhen 518055 China

**Keywords:** carbon nanotubes, environmental TEM, growth mechanism, interface interaction, symmetry breaking

## Abstract

The asymmetrical growth of a single‐wall carbon nanotube (SWCNT) by introducing a change of a local atomic structure, is usually inevitable and supposed to have a profound effect on the chirality control and property tailor. However, the breaking of the symmetry during SWCNT growth remains unexplored and its origins at the atomic‐scale are elusive. Here, environmental transmission electron microscopy is used to capture the process of breaking the symmetry of a growing SWCNT from a sub‐2‐nm platinum catalyst nanoparticle in real‐time, demonstrating that topological defects formed on the side of a SWCNT can serve as a buffer for stress release and inherently break its axis‐symmetrical growth. Atomic‐level details reveal the importance of the tube‐catalyst interface and how the atom rearrangement of the solid‐state platinum catalyst around the interface influences the final tubular structure. The active sites responsible for trapping carbon dimers and providing enough driving force for carbon incorporation and asymmetric growth are shown to be low‐coordination step edges, as confirmed by theoretical simulations.

## Graphical abstract

The asymmetrical growth of a single‐wall carbon nanotube from a sub‐2‐nm Pt catalyst is observed at atomic scale under reactive conditions. The axis‐symmetry of the SWCNT is broken due to the formation of step edges around the tube‐catalyst interface, which generates topological defects around the wall and results in variations of tube chirality.

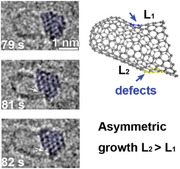

## 1 Introduction

Symmetry breaking is a pervasive phenomenon in nature, referring to many fields such as theoretical physics, bio‐chemistry, physic‐chemistry, and materials science.^[^ ^1^ ^]^ In the context of seeded growth of metal nanocrystals, asymmetrical growth has emerged as an efficient route to synthesize diverse anisotropic shapes and thus enhance their functions and applications.^[^ ^2^ ^]^ Such breaking of symmetry can be achieved through two main approaches: incorporating defects or stacking faults into the crystal lattice, or inducing differential growth using external species,^[^ ^3^ ^]^ such as etchants and reductants. In mechanistic details, both strategies affect the atom‐by‐atom arrangements of nanocrystals based on kinetics and thermodynamics,^[^ ^2^, ^4^ ^]^ with the former strategy involving intrinsic origins of defects and symmetry breaking. Similarly, for the synthesis of single‐wall carbon nanotubes (SWCNTs) that are nanoscale 1D chiral structures rolled up from monolayer graphene, the goal is to achieve their shape and size control for high‐end applications.^[^ ^5^ ^]^ Note that the main symmetry element for chiral SWCNTs is the axis of rotation, which may be changed by the local inclusion of a topological defect, such as separated pentagon and heptagon or their pairs. Recent research has shown that the chirality of SWCNTs is dictated by the atomic configuration of the catalyst nanoparticle,^[^ ^6^ ^]^ for example, enriched population of (12, 6) SWCNTs with sixfold symmetry were catalytically grown from a solid catalyst with the same structure symmetry.^[^ ^7^ ^]^ Hence, a change in the local atomic structure of catalyst^[^ ^8^ ^]^ is supposed to generate a local defect on the tube and then break the symmetry in the tube chirality, which can be either avoided for uniformity control or intentionally introduced for a specific asymmetric structure.

Without considering the effect of external forces or precursors on the CNT structure,^[^ ^9^ ^]^ theoretical calculations predicted that solid alloy catalysts favor complex growth kinetics by restructuring an asymmetrical CNT edge at the catalyst‐tube interface.^[^ ^10^ ^]^ While experimentally, Wei et al. found that ultralong few‐wall CNTs were highly symmetrical and uniform in the length‐direction,^[^ ^11^ ^]^ which is a requirement for the kinetically controlled growth of semiconducting CNTs.^[^ ^12^ ^]^ It is widely accepted that the structure of CNTs cannot be altered during growth. Therefore, uncovering the intrinsic origins of specific asymmetric structures in SWCNTs through solid evidence has become an intriguing subject of investigation. However, the atomic structure of catalysts and SWCNTs with a diameter <2 nm and the interfacial connections between them remain largely unexplored, especially during the course of the heterogeneous reactions involved in complex gaseous and elevated temperature conditions. By means of environmental transmission electron microscopy (ETEM), bending and irregular interlayer spacing have been observed in a 10 nm multi‐wall CNT,^[^ ^13^ ^]^ suggesting that it is possible to reveal the atomistic mechanisms at the catalyst‐tube interface that break the symmetry of a growing SWCNT.

Here, we report the direct atomic‐scale visualization of the symmetry breaking of a SWCNT during its dynamic growth from a sub‐2‐nm Pt catalyst particle, using in situ aberration‐corrected ETEM. Our findings reveal the formation of topological defects along the sidewall of the tube to release the stress, which alters the chirality of the tube. The origins of asymmetric growth are identified to be the formation of low‐coordinated step edges on the Pt nanoparticle, where the catalyst‐carbon interface evolves under reactive conditions. It is further confirmed that step edges assist the trapping of carbon atoms and their incorporation in the CNT by calculating the binding energies between carbon dimers and the catalyst.

## 2 Results and Discussion

### 2.1 Catalytic Nucleation of a SWCNT from a Sub‐2‐nm Pt Nanoparticle

**Figure 1:**
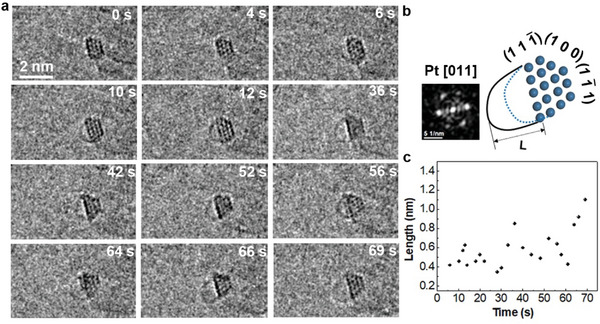
In situ observations of the nucleation of a SWCNT from a solid Pt catalyst at 550 ^o^C. a) Time‐sequential HRTEM images and b) scheme of the nucleation processes, during which the cap tangentially extended from the particle and fluctuated growth, the FFT inset in (b) shows the [011] zone axis of the particle. c) The maximum length of the embryo SWCNT fluctuates with time.

A Pt nanoparticle supported on MgO particle (**Figure** **1**a) was formed by in situ annealing of Pt/MgO in an ETEM system (see more details in Experimental Section). The sizes of the face‐centered cubic single‐crystal Pt nanoparticles were within 2 nm, indicating that the catalyst is composed of tens of Pt atoms (Figure S2, Supporting Information). This allows for the nucleation of SWCNTs with comparable radial dimensions to those produced by the chemical vapor deposition method. The series of HRTEM images and scheme in Figure 1a,b show the very early stages of SWCNT nucleation. The Pt nanoparticle retained its crystallinity for ≈70 s with negligible changes in size and morphology. In combination with the analysis of fast fourier transform (FFT), we confirm that our observation direction is always along the [011] zone axis of the faceted particle, which is tangentially connected to a short tube and exposed by {100} and {111} surfaces as shown in Figure 1b. A hemispherical cap lifted off at 36 s and only a very short extension was made until 69 s (from 0.8 to 1.1 nm). It is interesting to note that the nucleation of the CNT involves a fluctuation between tube elongation and shrinkage, as confirmed by the quantitative measurements in Figure 1c. These fluctuations of the short SWCNT and the relatively stable morphology of the particle suggest a weak relationship between the SWCNT and the Pt catalyst.

### 2.2 The Breaking of Symmetry During SWCNT Growth

**Figure 2:**
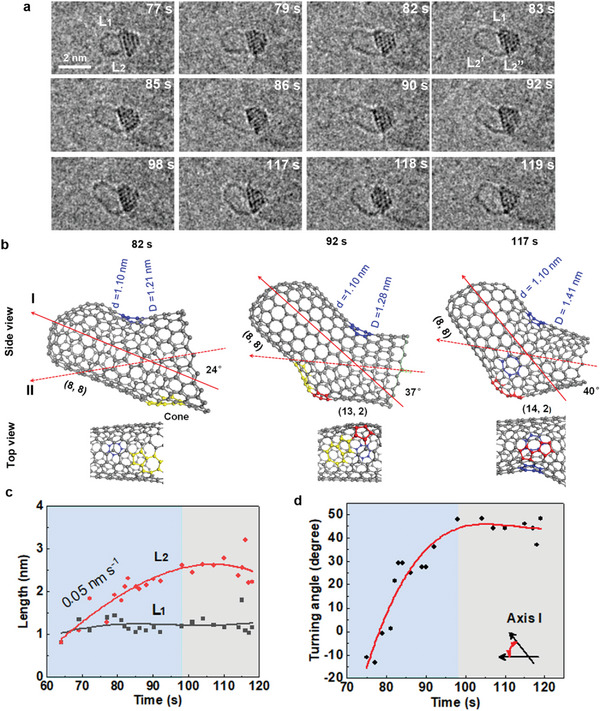
The transition from symmetrical to asymmetrical growth of a SWCNT. a) TEM images showing structural change of the growing SWCNT. b) Structural models of a typical asymmetric SWCNT formed during growth in (a) with STW defect pairs. The original tube diameter and the evolved one are defined as d and D, respectively. The pairs of (n, m) represents chirality indices. c) Lengths of opposite walls of the SWCNT as a function of time showing the different elongations in the projected image. L_2_ has two segments (L_2_’ and L_2_”) shown in (a), the average growth rate of the lower tube is ≈0.05 nm s^−1^ before 98 s. d) The turning angle of the original section of the tube as time. The tube growth and growth termination stages are highlighted by blue and grey colors, respectively, in (c,d).

A straight SWCNT is a cylindrical hexagonal network with chiral symmetry,^[^ ^14^ ^]^ for instance, the (8,4) and (12,6) SWCNTs have four and sixfold symmetry, respectively. The presence and the propagation of topological defects in the wall,^[^ ^15^ ^]^ may generate a change in the chirality of the cylindrical structure, thereby lowering the symmetry.^[^ ^16^ ^]^ In this study, with a constant carbon supply and growth temperature, the SWCNT with a diameter of ≈1.1 nm was continually attached to the particle while it incorporated more carbon atoms and grew longer in the period from 77 to 119 s (**Figure** **2**a), ruling out the effect of external forces. Assuming that the short tube grew perpendicular to the beam direction, the length of the SWCNT walls can be measured in the images. We call the wall at the top of the figure the upper with the other being the lower, and refer to their respective lengths as L_1_ and L_2_. Clearly, at 77 s the SWCNT is axially symmetric with the similar L_1_ and L_2_ values, but a few seconds later, the lower wall has extended while the length of the upper one has not changed, indicating a change in the growth of the tube from axial symmetry to asymmetry (Movie S1, Supporting Information).

As L_2_ increased with time, L_1_ remained ≈1 nm, when the ratio of L_2_ to L_1_ reached ≈1.75 at 82 s, the lower straight wall started to bend as indicated by the red arrow, a kink or junction acting as a buffer formed in the lower wall to release stress^[^ ^9^ ^]^ caused by its increasing length. Additionally, when the length ratio reached 2 at 83 s (Figure 2c), the bending angle increased from ≈22° to ≈45°, and then fluctuated between 20° and 45° during the following 15 s. This indicates that the defects around the junction propagate or transform with the asymmetric growth of the SWCNT.

In order to explain the structural change of the short SWCNT, atomic models of three typical SWCNTs in Figure 2a are shown in Figure 2b. The distortion of the tube wall is a kind of topological defect in the sp^2^ lattice of the SWCNT, consistent with the report that a convex defect on a tube wall during growth is purely a topological point defect or a bond rotation.^[^ ^17^ ^]^ This indicates that there are multiple Stone‐Thrower‐Wales (STW) defects consisting of five‐ and seven‐membered rings of carbon atoms, which evolve when the tube is under tension because of Joule heating caused by applying a bias voltage.^[^ ^18^ ^]^ Figure 2b shows possible topological defects such as an STW defect (yellow), single pentagon (blue), and single heptagon (red) defects formed in either the upper or lower walls. The defects can transform, gather, or propagate into new ones in the growing SWCNT, as confirmed by the different positions of the kink or junction, or the angle between the original (I) and new axes (II). During these changes, the chirality of the tube may change. For example, if the initial tube with a diameter (defined as d) of 1.1 nm is (8, 8), it could change to (13, 2) or (14, 2) as it grew, and the respective diameter (defined as D) changed to ≈1.3 and 1.4 nm. Clearly, STW defects lower the SWCNT symmetry^[^ ^19^ ^]^ or directly change its chirality^[^ ^18^ ^]^ by forming intramolecular junctions or kinks during its growth.

After these changes, the total length (L_2_) of the lower wall consists of the original segment L_2_’ and a new segment L_2_′. L_2_’ was measured to be ≈1.75 nm and remained almost unchanged from 83 to 98 s (Figure 2d), while L_2_″ increased slowly from 0.5 to 1.0 nm. At 98 s, L_2_ reached its maximum value which then slightly decreased after 117 s, meaning that the tube terminated growth along with an abrupt increase in its diameter, in agreement with our previous in situ study.^[^ ^20^ ^]^ By tracking the turning angle of the original section (L_2_’) of the tube, it increases gradually up to ≈60˚ at a rate of ≈3˚/s until the growth termination of the SWCNT (Figure 2d). We therefore conclude that this change is caused by structural changes in the SWCNT due to the breaking of its symmetry.

During the period of most rapid growth (64 s), the lower wall grows at an average rate of 0.05 nm s^−1^ (Figure 2c), compared to a much slower average rate of 0.005 nm ^−1^s in the early growth stage shown in Figure 1c. This suggests that new active sites may be exposed leading to faster carbon incorporation, since the external source has not changed. The faster or easier incorporation of excess/extra carbon atoms from the preferred active site is consistent with the report on the enhanced growth of multi‐wall CNTs from catalysts.^[^ ^13^ ^]^

### 2.3 Origin of the Asymmetric Growth of SWCNTs

**Figure 3:**
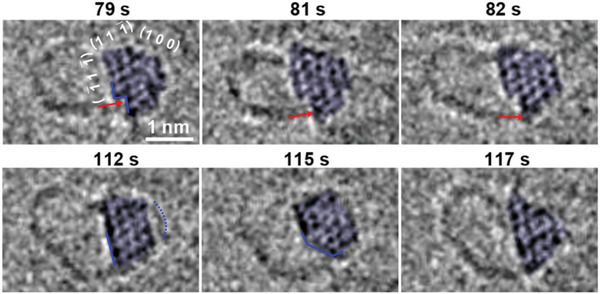
Assymmetric growth of a SWCNT from an atomic step site and the changes in the catalyst‐SWCNT interface formed by step movement.

To reveal the origin of the breaking of symmetry during the growth of SWCNTs, the catalyst‐tube interface was captured at the atomic scale (**Figure** **3**). It can be seen that a non‐Wulff structured Pt nanoparticle with {1 1 1} and {1 0 0} facets is first formed (Figure 1b), and a new monolayer of Pt partially covering the ($\bar{1}\;$1$\;\bar{1}$) surface then appears at 79 s, making more step edges exposed at the lower region of the Pt particle (as highlighted with the blue line), although the zone axis of the particle retains unchanged. After that, the connected SWCNT grew asymmetrically and the particle‐tube interface became less tangentially connected, compared to the connection during the early stage of SWCNT growth (Figure 1a). This indicates that low‐coordinated steps may be responsible for the breaking of the symmetry of the SWCNT, which takes as active sites for more carbon atoms incorporation into the downside wall. Note that the CNT lip moves from the inner step edge on the terrace to the outer step edge between 79 and 82 s (indicated by red arrows), increasing the tube diameter from 1.1 to 1.2 nm. This can be attributed to the weak catalyst‐tube connection. This is similar to a curved graphene edge, which is not bound strongly to a step^[^ ^21^ ^]^ or terrace,^[^ ^22^ ^]^ especially when a large force is applied. This line contact at the catalyst‐tube interface was also demonstrated by the inversed migration process of the SWCNT end in the period from 70 to 75 s (Figure S3, Supporting Information), during which a necked catalyst‐tube interface formed.

**Figure 4:**
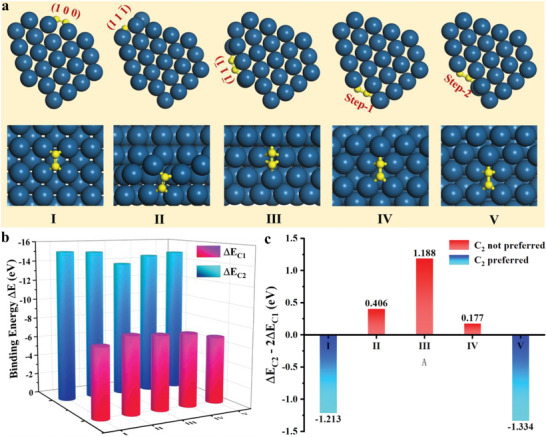
a) Optimized structures for the C_2_ stably adsorbed on (1 0 0) surface (site I), (1 1$\bar{1}$) surface (site II), ($\bar{1}$1$\bar{1}$) surface (site III) and two kinds of steps (site IV and V), b) Binding energies for the carbon monomer (ΔE_C1_) and carbon dimer (ΔE_C2_) adsorbed on five sites (from I to V), c) Energy driving force (ΔE_C2_–2ΔE_C1_) for the C_2_ formation on the five sites. The dark blue and yellow balls represent the Pt and C atoms.

As shown in Figure 2, SWCNT growth stopped at 98 s, from then the lower wall straightly covered on the ($\bar{1}\;\bar{1}\;$1) facet and the tube diameter increased to 1.4 nm. Especially, the highlighted terrace and step edges disappeared at 112 s, confirming that the step edges of the catalyst are correlated to the growth of SWCNTs. As more steps formed and evolved away from the original positions at 115 s, a longer lower wall is clearly observed, although the growth terminated at ≈98 s. This suggests that a surface contact of the tube and catalyst formed, different from the line contact mode during the fast asymmetric growth process before 82 s. Such a surface contact can also be seen in the upper wall when a layer of carbon covers on (1 1$\;\bar{1}$) and (1 0 0) surfaces, following a possible facet‐dependent anchoring mechanism,^[^ ^23^ ^]^ which was theoretically calculated in the next session that the driving force for the C_2_ formation on both (1 0 0) and (1 1$\;\bar{1}$) surfaces are preferred as shown in **Figure** **4**.

We attribute the formation and disappear of step edges or new terrace on the Pt surface to the atom rearrangement of the Pt catalyst driven by catalyst‐carbon interaction and thermal energy. We can see that a carbon layer highlighted by the dashed curve at 112 s was left as the underlying Pt atoms moved away, meanwhile the new monolayer terrace on Pt ($\bar{1}$1$\;\bar{1}$) disappeared with Pt atoms flowing into the adjacent surface. Except for the surface reconstruction of the facet of Pt particle, the step movements can also be observed in terms of the morphology change of the particle within only 5 s, which evolved from a nearly trapezoid morphology to an irregular hexagonal shape at 115 s and a triangular shape at 117 s. The above phenomena could be explained by the thermal instability of edge atoms of the Pt particle of the sub‐2‐nm Pt, especially with the adsorption of carbon atoms or covered by a graphene layer, such as the line contact of the tube and catalyst, which is similar to the discussion by Rao^[^ ^24^ ^]^ and the atomic evolution process of a large Ni particle by Hofmann.^[^ ^25^ ^]^ The atomistic details on Pt catalysts and the catalyst‐tube interaction represent the changes of surface or interface energies, in agreement with the process for the MWCNT growth, which is energy favored to initiate the growth from one side of the interface.^[^ ^13^ ^]^ In all, the stepped Pt catalyst‐tube interface is the origin for the asymmetrical growth of SWCNTs, which would be in turn modified and then extend the asymmetry.

### 2.4 Atomic Origin of the Asymmetric Growth of a SWCNT by Theoretical Calculations

It is clearly shown above that the formation of low‐coordinated step edges during SWCNT growth is the reason why asymmetry extended along the interface for the cylindrical structure. It is proposed that during the carbon atom incorporation, this active site triggers the continuous growth of SWCNTs. In order to confirm the activated growth of the SWCNT wall near the step edge, we calculated the adsorptions of both carbon monomer (C_1_) and carbon dimer (C_2_) on (1 0 0), (1 1$\;\bar{1}$), ($\bar{1}\;$1$\;\bar{1}$) surfaces and the two steps of the Pt catalyst (Figures S1, S4 and S5, Supporting Information) based on the density functional theory (DFT) calculations. The two types of steps are named as step‐1 and step‐2 with the latter one moving inward by removing one more row atoms of Pt ($\bar{1}$1$\;\bar{1}$) surface. The binding energies ΔE_C1_and ΔE_C2_ are calculated to evaluate the adsorption stability of the C_1_ and C_2_ on these different sites (Tables S1 and S2, Supporting Information). Where, ΔE_C1_ = E_C1/Pt_ − E_C1_ − E_Pt_ and ΔE_C2_ = E_C2/Pt_ − E_C2_ − E_Pt_. For the same surface (or the step) with more than one adsorption sites, the adsorptions with stronger binding energies are more energetically favored and therefore are the stable adsorptions. In detail, for the carbon monomer, the stable adsorption on the ($\bar{1}$1$\;\bar{1}$) and (1 1$\;\bar{1}$) surfaces is on the fcc and hcp site, respectively (Figure S4 and Table S1, Supporting Information). For the two steps, the fcc site near the step is more stable than other sites.

For carbon dimmer, only the steps have more than one adsorption sites for consideration, namely, the A and B sites. The calculated binding energies show that the C_2_ adsorbed on the A site of the step‐1 is more stable (Figure S5, Supporting Information), so that only A site is considered for step‐2. The optimized structure of the stable adsorption of C_2_ on the (1 0 0), (1 1 $\bar{1}$), ($\bar{1}$ 1 $\bar{1}$) surfaces and the two steps are presented in Figure 4a. Figure 4b shows the binding energies of the stable C_1_ and C_2_ adsorptions on the five sites. As the elemental building block for the SWCNT^[^ ^26^ ^]^ and graphene,^[^ ^27^ ^]^ the C_2_ formation on the catalyst can also be viewed as an essential reaction for the formation of the carbon network because both the nucleation and the continued growth always involve the formation of covalent C─C bond. The energy difference ΔE_C2_–2ΔE_C1_ is therefore defined as the driving force to evaluate whether the formation of C_2_ is preferred.^[^ ^28^ ^]^ The negative value indicates that the C_2_ is preferred while the positive one means not. The more negative the value is, the larger the driving force for the C_2_ formation. As shown in Figure 4c, it is clear that the C_2_ formation is preferred at the site I and V, that is, (1 0 0) surface and the step‐2. And, it is not preferred at the (1 1$\bar{1}$) and ($\bar{1}$ 1 $\bar{1}$) surfaces. Among them, the largest driving force for the C_2_ formation is on the step‐2, that is, the step moving inward, indicting the much faster growth of the SWCNT. For the step‐1, the driving force is slightly positive due to the relatively stronger adsorptions of C_1_. It should be noted that the similar negative driving force of C_2_ on (1 0 0) is consistent with the observation that carbon layers covered on it at 82 s in Figure 3, which is ineffective in incorporating more C_2_ due to the anchoring effect of the carbon layer.

### 2.5 Structural Evolution of Catalysts and Its Influence on SWCNT Chirality

In recent years, the “symmetry matching” route based on similar symmetries of catalysts and SWCNTs has been demonstrated effective in controlling nanotube chirality,^[^ ^7^ ^]^ however, there has been no direct evidence on the interfacial connections regarding the crystal symmetry of catalysts and SWCNTs. As for a given SWCNT with certain chirality, the changes of axis‐symmetry might be triggered by the evolved structure of catalysts, as well as different external sources, such as electrical current, mechanical stress or thermal energy.^[^ ^9^ ^]^ As for catalyst with symmetric morphology, we have reported a belt nucleation mechanism of SWCNTs from a truncated octahedron Pt nanoparticle.^[^ ^29^ ^]^ It has been very difficult to observe the catalyst‐tube interface and its evolution dynamics under HRTEM. The low elongation rate at a level of 0.05 nm s^−1^ and the aberration corrector enable us to follow the edge of SWCNT in contact with catalyst and evolution kinetics at atomic scale in this study. We found that the generated topological defects further induced the changes of the axis‐symmetry during the SWCNT growth process, and it is the first demonstration based on bottom‐up approach that carbon atoms are incorporated into hexagonal network from step edges along tube‐catalyst interface and would inversely influence the structure of catalysts.

As shown above, the formation of local edges in catalyst during catalytic growth of CNTs is the prerequisite of the asymmetrical growth of SWCNTs. While it is easy to occur since the catalyst evolution is believed one of the reasons for cap lift‐off. Even for a solid catalyst, the minute changes on the structure of catalyst might induce chirality changes, and the possibilities increase during the nucleation and growth process when its rate changes due to internal or external stress, *etc*. The step can be modified by carbon layer, which is then driven by the deformation of catalyst nanoparticles. It seems conflict that the nanoparticle with a solid crystalline structure being stable under carbon coverage. Our results imply that one needs to consider the stability of the catalyst active sites in real growth conditions for a perfect CNT synthesis, besides the morphology and symmetry of original catalyst particles. The criteria that determining SWCNT chirality can be the active sites of catalysts formed during the dynamical growth of a SWCNT, so it is predicted that a proper growth rate is necessary for remaining CNT symmetry.

## 3 Conclusion

In summary, we directly observed the breaking of the axis‐symmetry of SWCNTs grown from sub‐2‐nm Pt catalysts under reactive conditions. During the asymmetrical growth, stress was accumulated on one side of a SWCNT and finally released by changing the tube axis with a maximum rotation angle of ≈60˚, and forming series of topological defects around tube junction. The diameter and chirality of the SWCNT were also found to vary. The underlying mechanism for the structural evolution has been revealed as the catalyst‐SWCNT interaction, which triggers the formation of step edges locally on Pt particle around the interface. The active sites are confirmed by DFT calculations to preferentially trap dimers and provide large enough driving force for the additional carbon incorporation.

## 4 Experimental Section

### Catalyst Preparation

The Pt/MgO catalysts were prepared by an impregnation method using H_2_PtCl_6_.6H_2_O solution as Pt precursor, followed by calcination at 400 °C for 1 h, the loading of Pt is ≈0.5 wt.%. The sample was dispersed in ethanol and drop‐casted on a micro‐heater from DENSsolutions B.V. After drying at room temperature, the sample was put in an ETEM (FEI Titan 80–300ST) system operated at 300 kV for investigation.

### In Situ ETEM Experiment

The in situ annealing conditions were optimized to ensure the formation of Pt nanoparticles suitable for CNT growth. Pt nanoparticles with sizes <2 nm were formed in vacuum at 550 °C on the MgO support, which were reduced under hydrogen atmosphere for 1 h. After that, hydrogen was pumped away. Ethanol vapor with a pressure of (3–5) × 10^−4^ mbar was used as a carbon source to initiate the nucleation of SWCNTs. During the above process, the structures and evolution dynamics of particles and SWCNTs were observed and recorded using a Gatan US1000 camera with an exposure time of 0.5 s.

### Theoretical Calculations

The DFT calculations were performed using the Vienna Ab initio Simulation Package (VASP).^[^ ^30^ ^]^ The Projected Augmented Wave (PAW)^[^ ^31^ ^]^ pseudopotential approach and the Perdew–Burke–Ernzerhof generalized gradient approximation (PBE‐GGA)^[^ ^32^ ^]^ were employed to describe the electrons‐nuclei interactions and the exchange‐correlation interactions, respectively. The DFT‐D3 dispersion correction was used to describe the van der Waals interaction.^[^ ^33^ ^]^ The cutoff energy of the electron wave function was set to 400 eV. The energy and force convergence for the geometry optimization were set at 10^−4^ eV and 0.01 eV Å^−1^, respectively. As shown in Figure S1 (Supporting Information), a supercell of 5.55 Å × 30 Å × 30 Å is built to mimic the experimental Pt catalyst with (1 0 0)/(2 0 0), (1 1 $\bar{1}$), ($\bar{1}$ 1 $\bar{1}$) surfaces and the step. The Monkhorst‐Pack k‐mesh of 5 × 1 × 1 is therefore used for the Brillouin zone's sampling.

## Conflict of Interest

The authors declare no conflict of interest.

## Supporting information

Supporting Information

Supplemental Movie 1

## Data Availability

The data that support the findings of this study are available in the supplementary material of this article.
