# Supplementary material for: Breaking the Axis‐Symmetry of a Single‐Wall Carbon Nanotube During Its Growth
Source: Adv Sci (Weinh). 2023 Oct 28;10(36):2304905. doi: 10.1002/advs.202304905 (PMC10754088; doi:10.1002/advs.202304905)
Supplement: Supplementary file 1 — Supporting Information [file ADVS-10-2304905-s002.pdf]

ADVANCED SCIENCE
Open Access

# Supporting Information

for *Adv. Sci.*, DOI 10.1002/advs.202304905

Breaking the Axis-Symmetry of a Single-Wall Carbon Nanotube During Its Growth

*Lili Zhang*, Ziwei Xu, Tian-liang Feng, Maoshuai He, Thomas Willum Hansen, Jakob Birkedal Wagner, Chang Liu* and Hui-Ming Cheng*

# Supporting Information

## **Breaking the Axis-Symmetry of a Single-Wall Carbon Nanotube During Its Growth**

*Lili Zhang,* [‡]* *Ziwei Xu,*[‡] *Tian-liang Feng, Maoshuai He, Thomas W. Hansen, Jakob B. Wagner, Chang Liu,* Hui-Ming Cheng*

L. Zhang, C. Liu, H. M. Cheng
Shenyang National Laboratory for Materials Science
Institute of Metal Research, Chinese Academy of Sciences
72 Wenhua Road, Shenyang 110016, China
E-mails: zhangll@imr.ac.cn, cliu@imr.ac.cn

Z. Xu, T. Feng
School of Materials Science and Engineering
Jiangsu University
Zhenjiang 212013, China

M. He
College of Chemistry and Molecular Engineering
Qingdao University of Science and Technology
Qingdao 266042, China

T. W. Hansen, J. B. Wagner
DTU Nanolab
Technical University of Denmark
2800, Fysikvej, Kongens Lyngby, Denmark

H. M. Cheng
Institute of Technology for Carbon Neutrality
Shenzhen Institute of Advanced Technology, Chinese Academy of Sciences
1068 Xueyuan Road, Shenzhen 518055, China

‡ L. Z. and Z. X. contributed equally to this work.

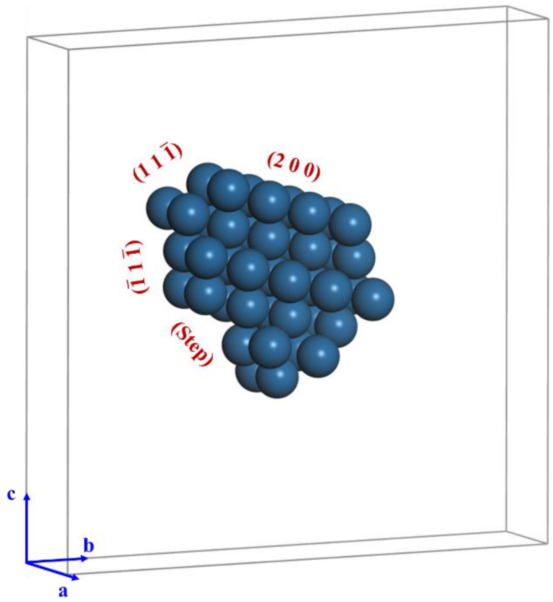


**Figure S1.** Supercell of the Pt catalyst for DFT calculations with a=5.55 Å, b=30 Å, c=30 Å.

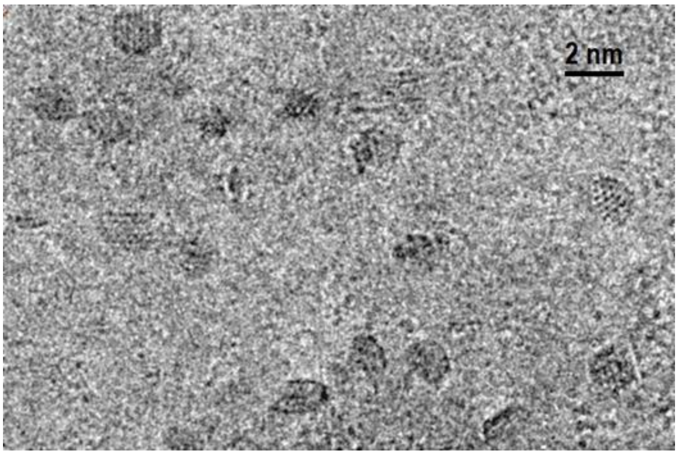


**Figure S2**. Sub-2-nm Pt nanoparticles formed on MgO by *in situ* annealing Pt/MgO precursors in ETEM.

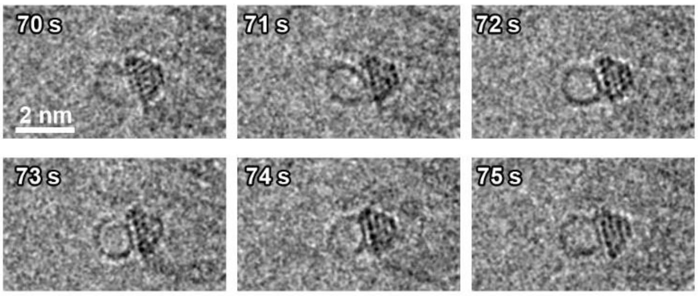


**Figure S3.** The SWCNT growth halted along with a necked interface evolution.

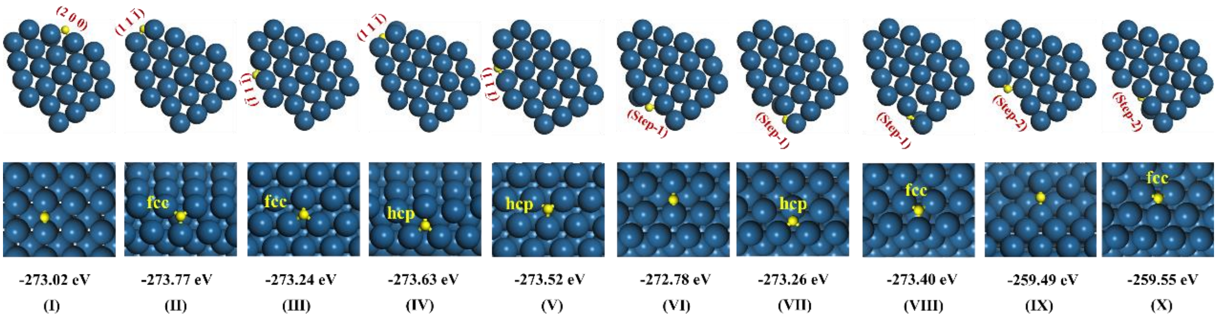


**Figure S4.** Side views (upper panel), top views (bottom panel) of the optimized structures of the C monomer adsorbed on ten different sites of the Pt catalyst as well as their energies. (I) (2 0 0) surface, (II) fcc site on (1 1 $\bar{1}$) surface, (III) fcc site on ($\bar{1}$ 1 $\bar{1}$) surface, (IV) hcp site on (1 1 $\bar{1}$) surface, (V) hcp site on ($\bar{1}$ 1 $\bar{1}$) surface, (VI-VIII) different sites on step-1, (IX-X) different sites on step-2. The dark blue and yellow balls represent the Pt and C atoms.

**Table S1.** Binding energies of the optimized structures of C monomer adsorbed on ten different sites of the Pt catalyst.

| Site | I | II | III | IV | V | VI | VII | VIII | IX | X |
|---|---|---|---|---|---|---|---|---|---|---|
| $\Delta E_{C1}$[eV] | -6.962 | -7.721 | -7.182 | 7.573 | -7.465 | -6.729 | -7.209 | -7.344 | -6.665 | -6.725 |

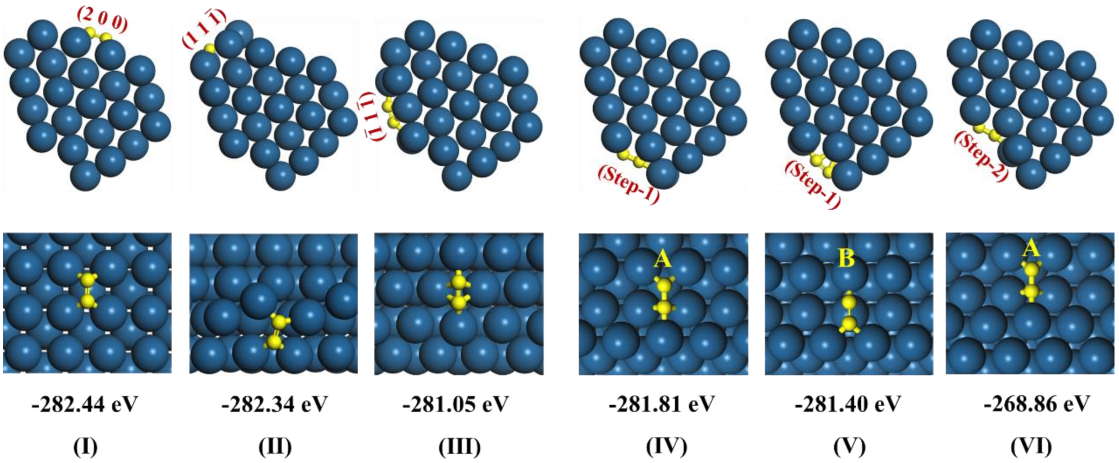


**Figure S5.** Side views (upper panel), top views (bottom panel) of the optimized structures of the C dimmer adsorbed on the six different sites of the Pt catalyst as well as their energies. (I) (2 0 0) surface, (II) (1 1 $\bar{1}$) surface, (III) ($\bar{1}$ 1 $\bar{1}$) surface, (IV) A site on the step 1, (V) B site on the step-1, (VI) A site on step-2. The dark blue and yellow balls represent the Pt and C atoms.

**Table S2.** Binding energies of the optimized structures of C dimmer adsorbed on six different sites of the Pt catalyst.

| Site | I | II | III | IV | V | VI |
|---|---|---|---|---|---|---|
| $\Delta E_{C2}$ (eV) | -15.14 | -15.04 | -13.74 | -13.56 | -14.10 | -14.78 |
